# Event-related (de)synchronization and potential in whole vs. part sensorimotor learning

**DOI:** 10.3389/fnsys.2023.1045940

**Published:** 2023-03-21

**Authors:** Juan J. Mariman, Trinidad Bruna-Melo, Rosario Gutierrez-Rodriguez, Pedro E. Maldonado, Pablo I. Burgos

**Affiliations:** ^1^Department of Physical Therapy, Faculty of Medicine, Universidad de Chile, Santiago, Chile; ^2^Cognition and Sensorimotor Behavior Lab, Department of Physical Therapy, Faculty of Arts and Physical Education, Universidad Metropolitana de Ciencias de la Educación, Santiago, Chile; ^3^Nucleus in Wellbeing and Human Development, Education Research Center (CIE-UMCE), Universidad Metropolitana de Ciencias de la Educación, Santiago, Chile; ^4^Motor Learning and Neurorehabilitation Lab, Faculty of Medicine, Universidad de Chile, Santiago, Chile; ^5^Department of Neuroscience, Faculty of Medicine, Universidad de Chile, Santiago, Chile; ^6^Neuro Systems Lab, Faculty of Medicine, Universidad de Chile, Santiago, Chile

**Keywords:** ERP, ERD/ERS, motor learning, skill learning, whole-part

## Abstract

**Background:**

There are different ways to learn a sensorimotor task. This research focuses on whole versus part learning in a complex video game that involves sensorimotor adaptations and skill learning. The primary aim of this research is to compare the changes in (1) event-related potentials (ERP) and (2) Alpha and Beta event-related desynchronization/synchronization [ERD(S)] of EEG between whole and part practice protocols.

**Materials and methods:**

18 Healthy young participants practiced for 5 days a video game with distorted kinematic (advancing skill) and dynamic features (shooting skill) to test the ability to combine sensorimotor skill components learned modularly (part learning, 9 participants) or combined (whole practice, 9 participants). We examined ERP and ERD(S) in EEG channels in the baseline test (day 1) and the retention test (day 5), dissociating epochs with advancing or shooting. We focus the analysis on the main activity of ERP or ERD(S) in different time windows.

**Results:**

In the advancing epochs (distorted kinematic), both groups showed a decrease in time for ERP and an increase in Beta ERD activity in central and posterior channels. In the shooting epochs (distorted dynamic), the Whole group showed a decrease in time for ERPs in anterior and central-posterior channels. Additionally, the shooting ERS in the Beta band decreases within sessions in central channels, particularly for the Part group.

**Conclusion:**

Neural correlates of kinematic and dynamic control [ERP and ERD(S)] were modulated by sensorimotor learning, which reflects the effect of the type of practice on the execution and the evaluation of the action. These results can be linked with our previous report, where the simultaneous practice of kinematic and dynamic distortions takes advantage of the motor performance on retention tests, indicating a more automatic control for the whole practice group.

## 1. Introduction

The classic definition of motor learning considers that there must be a relatively permanent behavior change (Schmidt et al., 2019) since the substrate of the changes occurs in brain networks that we cannot directly observe. Acquiring a new sensorimotor skill involves different processes that compose it, such as spatial perception of the body concerning the environment, kinematic and kinetic adaptations, and use-dependent skill acquisition (speed, precision, optimization, variability reduction) (Krakauer and Mazzoni, 2011).

It is well-known that in addition to the amount of practice, there are conditions that favor or interfere with motor learning, called practice conditions (Schmidt et al., 2019). In this study, we will focus on the correlation of brain activity associated with the practice condition called Whole and Part practice (Fontana et al., 2009; Schmidt et al., 2019). The Whole training corresponds to practice without divisions of a motor task (e.g., driving a manual car), unlike the training by parts where the task is segmented, fragmented, or simplified to be learned (Magill and Anderson, 2017).

The literature on Whole and Part practice highlights each learning form’s advantages and disadvantages. There is no consensus yet on whether it is better to train different types of task components or sensorimotor skills totally or in parts (Fontana et al., 2009). For the Whole practice, the advantage of precise temporal coordination between effectors and ongoing task components has been described. Its disadvantages have been reported concerning the initial difficulty and the lack of focus on the main difficulties of the task. The advantages and disadvantages of Part practice emerge from the previous description. Although coordination issues are recognized, this type of practice simplifies learning and focuses on some task components, outperforming the Whole learning in some cases (Klein et al., 2012). In general, it is accepted that the advantages or disadvantages of training the whole task or in parts will depend on the amount in which the parts of these tasks interact for the whole task and the number of perceptual components of the task (Schmidt et al., 2019). In this sense, the kinetic and kinematic components of the task could be trained separately or integrated. Recently, we tested if the integrated training of kinetic and kinematic adaptations overcame the training of these components in parts (Burgos et al., 2018). Our results confirm that the integration of task components demands instances of coordination, but the time cost for Part practitioners to integrate is low. Could such differences be related to the degree of connectivity between relevant cortical areas? In the same study, we showed that visual and motor cortical domains are differentially connected depending on the type of practice, being more independent when the integration of kinetic and kinematic components was carried out from the beginning of practice (in the case of Whole practice). Therefore, the amount of connectivity indexes the coordination of task components and performance. While these results emphasize the role of cortical connectivity as a mechanism to integrate neural operations that support learning, differences between Whole and Part practice could also come from neural processing in local regions shown by ERP and ERD(S).

Previous reports link the modulation of neural signals in the time and frequency domains with specific aspects of motor control. In the time domain, it has been described that movement-related cortical potentials (MRCPs) are associated with planning or preparation (readiness potential, RP), execution (motor potential), and control of performance (movement-monitoring potential, MMP) (Dremstrup et al., 2013). Several factors could influence these potentials, such as force exerted, speed, and precision of movement or learning (Shakeel et al., 2015). Evidence shows a change in the motor event-related potential (motor-ERP) during the motor learning process (Staines et al., 2002; Hill and Raab, 2005; Allami et al., 2014). Regarding erroneous actions or incorrect responses, it has been described two components of ERP after the error has occurred. The early component is called error-related negativity (ERN), about 0–100 ms after, and the later component is a positivity peak (known as Pe), about 150 to 200 ms after (Falkenstein et al., 1991, 2000; Gehring et al., 1993; Vocat et al., 2011). Anguera et al. (2009) reported that the error-related negativity (ERN) is higher during trials, with more error for frontocentral electrodes when participants must adapt to kinematic perturbations. Similar results are described for reaching movements during prism adaptation, where ERN from frontal locations (close to FC location in 10–20 EEG coordinates) indexes the movement accuracy (Vocat et al., 2011; MacLean et al., 2015) being lower the evoked negative response as the reaching error decrease.

On the other hand, the analysis of the movement-related activity in the frequency domain has been extensively characterized. For both the Alpha (8–12 Hz) and Beta (13–30 Hz) frequency range, a modulation of spectral power is described, characterized by a decrease in power during the execution of the movement (ERD), as well as a recovery of the post-completion activity (ERS). However, the interpretation of these two phenomena is different. In motor tasks, ERD in the Alpha range for central electrodes has been associated with the general demands of the task, its attentional load, and sensory processing (especially for the mu rhythm) (Kilavik et al., 2013). In the case of frontal Beta ERD, it is associated with the activation of motor networks due to the drop of Beta power that lasts until the end of the movement (Wheaton et al., 2009). Also, Beta ERD is present during visuomotor reaching tasks under normal or distorted kinematic conditions (Tatti et al., 2021), during motor imagery (Nakagawa et al., 2011), and movement observation (Koelewijn et al., 2008). This evidence ratifies that this oscillatory phenomenon is implicated in the planning and execution of movement. After ERD, the Beta activity recovers to pre-movement magnitude (ERS), whose origin could extend to the sensory, motor, and frontal regions involved in performance evaluation and movement recalibration (Kilavik et al., 2013). Beta ERS is associated with motor adaptation under distorted conditions (Tan et al., 2014; Torrecillos et al., 2015), where the ERS magnitude is related to the movement error. Thus, examining the related-neural activity (ERP and ERD-ERS) to the movement can help to explore the origin of the differences in the types of practice.

In this research, we seek to elucidate the effect of the type of practice (whole vs. parts) when we need to adapt the kinetic and kinematic control of effectors, focusing our analysis on the neural correlates in time and frequency of the activity of EEG channels. We hypothesize that the advantage of coordination that confers the whole practice is reflected by a less but better time-tuned neural activity in critical scalp regions.

## 2. Materials and methods

### 2.1. Participants

Eighteen participants were included in the experimental learning protocol (mean: 23 years, range: 18–37 years), randomly assigned to the whole (9 participants) and Part (9 participants) groups. All participants were right-handed males without neurological or psychiatric illness with normal or corrected vision. They all had previous experience with video games and practiced an average of 4 h per week (range: 1–8 h per week).

The Ethics Committee of the Faculty of Medicine at the University of Chile approved the study. All the participants gave written informed consent following the Declaration of Helsinki. The current study is a complementary analysis of the original report of our group (Burgos et al., 2018).

### 2.2. Video game task

**FIGURE 1:**
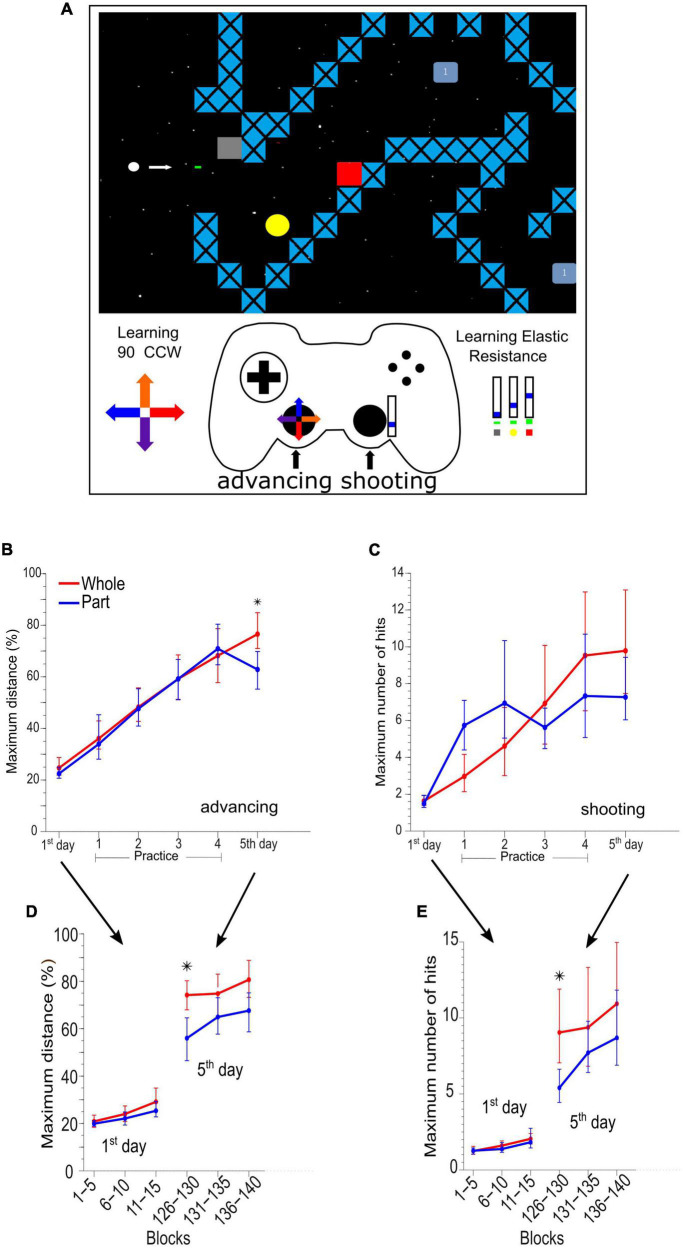
Adaptation and skill learning of advancing and shooting in the videogame task. **(A)** Example of a videogame route where the main character (white dot) has two purposes, first to advance as much as possible without colliding and second to destroy the enemies (yellow circle, red square, and light blue square) by means of bullets. The left stick of the gamepad controls the motion of the character, and it has a kinematic distortion of 90°counterclockwise. The right stick of the gamepad controls the bullet generation with an augmented elastic resistance (dynamic distortion). **(B)** Maximum advance in the path by group and by sessions in 5 days of practice and assessment. **(C)** Maximum hits destroying enemies by groups and sessions. **(D)** Maximum advance every 5 blocks of 90 s on days 1st and 5th. **(E)** Maximum hits destroying enemies every 5 blocks of 90 s on days 1 and 5. *Means significant difference between groups.

The task was a custom-made video game programmed in Python (Python Software Foundation, version 2.6) where the participants had to learn to advance a circular cursor in 10 different path configurations, controlling the cursor position to avoid colliding with stationary and moving obstacles and simultaneously destroy these obstacles by shooting bullets. The participants were instructed to “advance as much as you can” and “destroy obstacles as much as you can.” When the participants collided with any objects, the game was reset from the beginning of a new path selected randomly from the set [Figure 1A and details in Burgos et al. (2018)].

The cursor advance required learning a new kinematic rule (kinematic adaptation) with the left hand moving the left stick of a gamepad (dual action*™* Logitech). The movements produced by the left thumb on the left analog stick were rotated on the screen by 90 degrees counterclockwise (Figure 1A bottom left, color arrows in the cross-over left lever of the gamepad and on the path). The participants also had to learn a new dynamic rule (dynamic adaptation) to destroy obstacles by manipulating the gamepad with their right thumb. An elastic resistance was added to the right analog stick of the gamepad by employing a rubber band of 1.4 × 13 cm (TheraBand Silver resistance band). The participants needed to learn to displace the stick within three position ranges, 0–15, 15–30, and 30–45%, of the whole range of movement in the upward direction of the right analog stick to destroy three different types of obstacles (Figure 1A, bottom right). The shot occurred when the lever returned to the center (switch) and then returned to some of the three ranges by at least 360 milliseconds.

### 2.3. Experimental groups

We established two experimental learning groups trained to perform a visuomotor task that had an advancing with a kinematic perturbation and shooting with a dynamic perturbation.

The Whole group had to practice and learn both components simultaneously (whole practice), and the Part group practiced and learned each motor component in separate sessions (part practice).

### 2.4. Learning protocol and assessments

Both previous groups were exposed to an initial session consisting of 15 blocks of 90 s of integrated practice (combined) to obtain a baseline measurement with the kinematic and dynamic perturbations (1st-day). The 1st-day session (Figure 1B) was followed by a daily session for 4 days, in which the participants in the Whole group continued to practice advancing and shooting simultaneously (20 blocks of 90 s on day 1, and 30 blocks on days 2 to 4). Contrastingly, the Part group practiced first advancing and then shooting (or vice versa; randomly balanced, i.e., 10 blocks of advancing and 10 blocks of shooting on day 1, and 15 and 15 blocks on days 2 to 4). On day five, all the participants were evaluated during a combined advancing and shooting session (5th day).

### 2.5. Behavioral metrics

Advancing (kinematic component) learning (Figures 1B, D) was evaluated as the maximum advanced distance of each block. The shooting (dynamic component) learning was evaluated as the maximum number of hits achieved in the different attempts within each block of 90 s (Figures 1C, E). We analyzed the advancing and shooting performance by comparing sets of 15 game blocks (complete session). To obtain a detailed analysis, we also compared groups of 5 continuous game blocks on day 1 (blocks 1–5, 6–10, and 11–15) and day 5 (blocks 125–130, 131–135, and 136–140) respectively (See Figures 1B, C).

### 2.6. Electroencephalography acquisition and data preprocessing

Continuous EEG signals were recorded during days 1 and 5 of the learning protocol by employing an ActiveTwo BioSemi electrode system using 32 scalp electrodes plus six extraocular electrodes and two electrodes in both mastoids digitized at 512 Hz. The output impedance of each active sensor was < 1 Ω according to the BioSemi Company’s specifications.^1^

The acquired EEG data were imported using the EEGLAB toolbox^2^ with a bilateral mastoid reference to be processed in MATLAB. Continuous data were filtered with a high-pass filter at 1 Hz and a low-pass filter at 90 Hz; a notch filter at 50 Hz was included to remove the line noise using the function “cleanline.”^3^ The events were identified using our MATLAB algorithm, which detected the onset of joypad displacement executed by the left thumb (advancing events) or the right thumb (shooting events) based on position and velocity criteria. Only the events without a temporal overlap of other events, 0.5 s before and 0.3 s after were selected. For EEG analysis, we collected epochs of 3 s, which were extracted from −1.5 to 1.5 s regarding the onset of movement (advancing and shooting epochs). Epochs with artifacts in the channels were deleted with an automated method in EEGLAB that takes into account extreme values of potential (μV), data improbability, and kurtosis of potential (Delorme et al., 2007). Then the data were visually inspected to check for noise artifacts. The number of clean epochs used per session and participants for advancing and shooting was 84.

Each dataset was further analyzed with an Adaptive Mixture Independent Component Analysis (ICA) of the data for decomposition into source-resolved activities.^4^ The data were then re-referenced with an average montage. Finally, the equivalent current dipole location was computed using a Boundary Element Model of the MNI head model with the dipfit toolbox of EEGLAB. Independent components with a location or activity related to eye movements, blinks, cardiac or muscular noise were removed. After the rejection, the percentage of kept Independent components was 53.9% (6.8 SD).

### 2.7. EEG metrics

Based on previous analysis (Burgos et al., 2018), we analyzed all the channels and estimated ERPs and ERDs. Only the channels that had significant differences between the groups and/or sessions are shown in the results. First, the ERPs were estimated by computing the average voltage amplitude for epochs grouped by condition (advancing or shooting) and sessions (integrated test of day 1 and day 5). Also, for each epoch, we normalized the voltage time series by subtracting a baseline value (period from −1.5 to 0 s).

Second, the ERD(S) was estimated by computing the power spectrum over a sliding window and averaging across the epochs. We applied a sinusoidal wavelet transform (short-time DFT) with a 2 s epoch length divided into 200 temporal segments to obtain the power spectrum. The lowest frequency examined was 3 Hz with three cycles, and the maximum frequency was 50 Hz with ten cycles for wavelet transformation. A baseline from −1.5 to 0 s was used to normalize each frequency bin. The distance between the two output frequency bins was 0.5 Hz. ERD(S) was interpreted for frequency bands (Alpha, 8–12 Hz; Beta, 12–30 Hz).

For all time series analyses (ERP and ERD), time 0 s corresponded to the onset of thumb movement in epochs of advancing or shooting as appropriate.

Different temporal windows were used in relation to the main activity of central and posterior channels in ERP or ERD(S) signals. The average of these time windows was used for statistical analysis.

### 2.8. Statistics

Behavioral, ERP, and ERD(S) metrics were statistically compared in Matlab (Matworks Inc.) and eeglab toolbox. The analysis was for 2 factors. The first factor was unpaired for Groups (Whole vs. Part). The second factor was paired for sessions (day 1 vs. day 5 integrated test). For behavioral data, the 2 way ANOVA for repeated measures was used with a Sidak test as a *post hoc*, particularly the interaction effect, to determine group differences in learning. For ERP and ERD(S) we used *T*-test with 2,000 permutations of subjects per group with the false discovery rate (fdr) as a *post hoc* for sessions (paired) and groups (unpaired). For the interaction of sessions and groups, we also used a 2 way ANOVA with permutations and fdr. In the Supplementary material (Supplementary Figures 1, 2) show the statistical results of EEG analysis without the fdr corrections to see the general trends of the EEG variations.

## 3. Results

### 3.1. Whole practice temporarily overcame part practice after training

Previously (Burgos et al., 2018), we described the behavioral effect of Whole vs. Part practice extensively. Here we summarize such results. The Whole group outperformed the Part group on the advancing skill [*F*_(1_,_16)_ = 8.074, *p* = 0.012] in the integrated test respecting the baseline test (Figure 1B). In the analysis by a set of 5 blocks (Figure 1D), the Whole group showed a significantly greater performance than the Part group in the first set of blocks 126 to 130 [*F*_(1_,_16)_ = 9.557, *p* = 0.007]. Still, these differences quickly subsided in the following sets (block 131 ahead).

In the shooting skill, there is no difference in the integrated test considering all blocks (Figure 1C). When we grouped the data in a set of 5 blocks (Figure 1E) we observed a significant outperformance of the Whole group on the first set of the integrated test [*F*_(1_,_16)_ = 7.934, *p* = 0.012] although, it was equated by the Part group in the successive blocks. The statistical power of behavioral whole vs. part differences can be checked in Supplementary Table 1.

These results demonstrated that the part practice was not immediately integrated but it was quickly coordinated within the same session.

### 3.2. Advancing ERPs in central and posterior channels decrease within days, with group differences at different times

**FIGURE 2:**
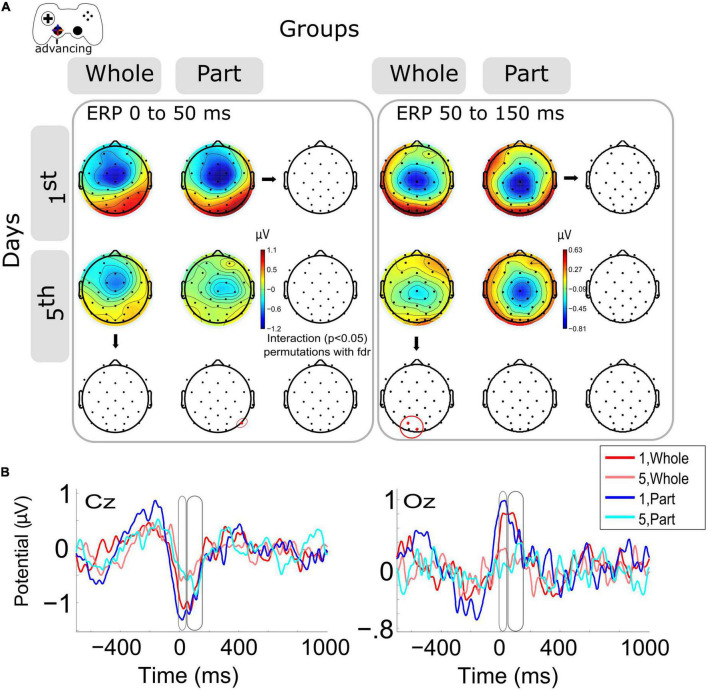
Modulation of the event-related potentials (ERP) activity by days and groups during advancing motion. **(A)** The topography of all channels is represented in the scalp map at the time window of 0 to 50 ms and from 50 to 150 ms, as motor activity of advancing in the videogame. In red dots and red ellipses are depicted the significant differences, in this case for the within-session variation for the Part group (0 to 50 ms) and for the Whole group (50 to 150 ms). **(B)** ERPs per session and groups of channels Cz and Oz. Time 0 ms is the motion onset, 1st = baseline test, 5th = integrated test.

When the ERP modulation within days was analyzed within days 1st and 5th Figure 2A left), we observed a significant decrement (*p* < 0.05) of ERP amplitude (μV) around the advance motion period (0 to 50 ms) for the P8 channel in the Part group. The same trend was observed in central and posterior channels in both groups without significant differences (Supplementary Figure 1A). At the same time, we observed a significant decrease (*p* < 0.05) of ERP amplitude (μV) in the period after the advance motion onset (time window from 50 to 150 ms) for posterior channels (Oz, PO3) in the Whole group. The same decrease was observed in the Part group without significant differences (Supplementary Figure 1B). The interaction was not significant. The topographies in this time window show that the Part group has a longer and more negative ERP amplitude, keeping the activity of the previous time window (0–50 ms).

Representative channels for ERPs (Cz, Oz) were selected to show the complete epoch in Figure 2B. The highest variation in voltage occurs at the onset of advancing motion (time 0 ms).

### 3.3. Shooting ERPs in anterior and central-posterior channels decrease within sessions about the motion feedback, with group differences

**FIGURE 3:**
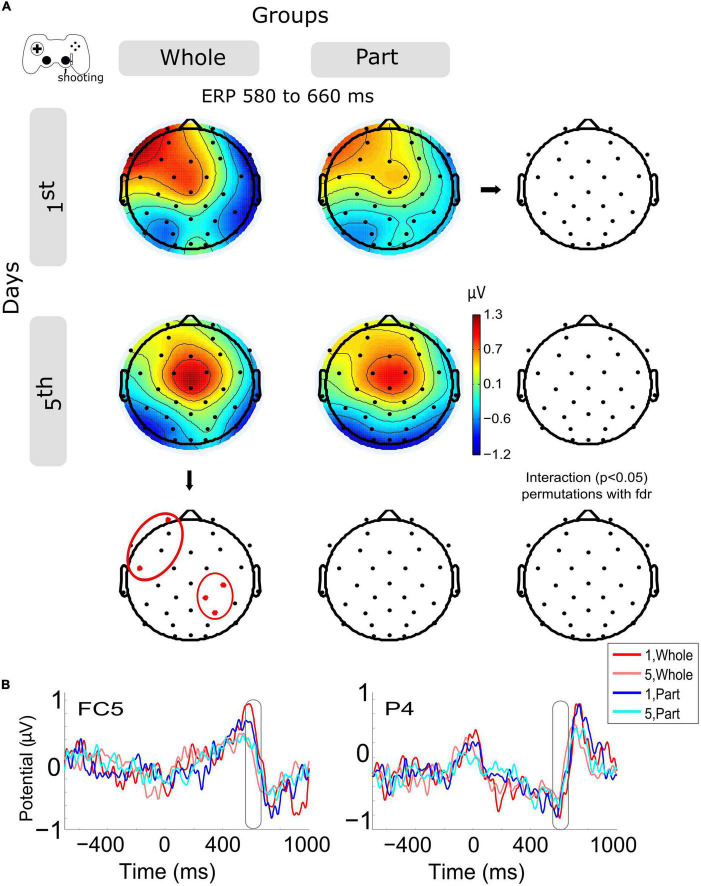
Modulation of the event-related potentials (ERP) activity by days and groups during shooting motions. **(A)** The topography of all channels is represented in the scalp map at the time window of 580 to 660 ms, as a monitoring activity of the shooting consequences in the videogame. In red dots and red ellipses are depicted the significant differences, in this case for the within-session variation for the Whole group. **(B)** ERPs per session and groups of channels FC5 and P4. Time 0 ms is the motion onset, and about 400 ms is the motion offset. 1st = baseline test, 5th = integrated test.

The ERPs of shooting epochs (Figure 3B) show the main change in voltage amplitude with the motion feedback (about 600 ms, checking if the bullets destroyed the enemies in the videogame) after the offset of shooting motion (about 400 ms). The amplitude variation within sessions (Figure 3A) shows a significant decrease in left anterior channels (FP1, FC5) and in right central-posterior channels (C4, CP2, P4) for the Whole group. The same variation was observed in the Part group without significant results (Supplementary Figure 1C). The group and day interaction was not significantly different.

### 3.4. Advancing ERD in the beta band increases within sessions in the central posterior channels after the motion onset

The time-frequency activity for Alpha (8–12 Hz) and Beta (12–30 Hz) band was examined in 2 temporal windows for advancing epochs, 0–400 and 400–800 ms. The Alpha activity increases in central and posterior channels within the session for both groups without significant differences (not shown in the Figures).

**FIGURE 4:**
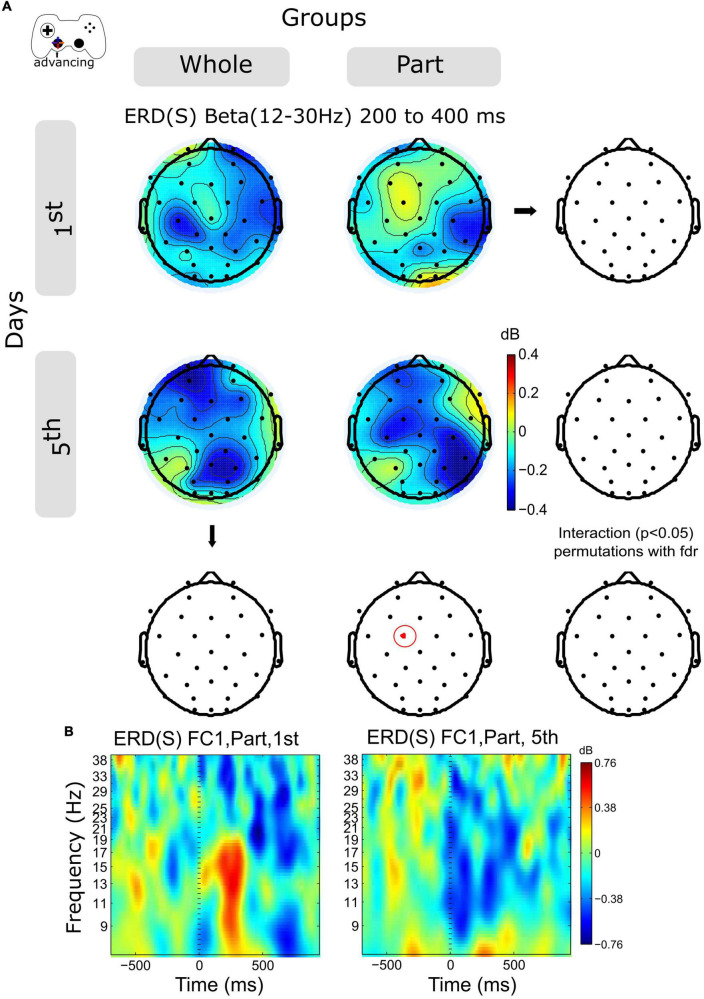
ERD/ERS by days and by groups during advancing. **(A)** The topography of power (dB) in all channels is represented in the scalp map at the time window of 200 to 400 ms. In red dots and red ellipses are depicted the significant differences. **(B)** FC1 time-frequency plots of advancing epochs of Part group in days 1st and 5th. Time 0 ms motion onset. 1st = baseline test, 5th = integrated test.

The ERD activity of the Beta band shows an increase in central and posterior channels within sessions in both groups (Figure 4A and Supplementary Figure 2A), however, this variation was significant only for the Part group in channel FC1, in the time window 200–400 ms (Figures 4A,B).

### 3.5. Shooting ERS in the beta band decreases within sessions in central channels after the motion offset

The time-frequency activity for Alpha ERD decreases in central and posterior channels within the session for both groups in the time windows from 0 to 400 ms, without significant results for any factor (not shown in Figures).

**FIGURE 5:**
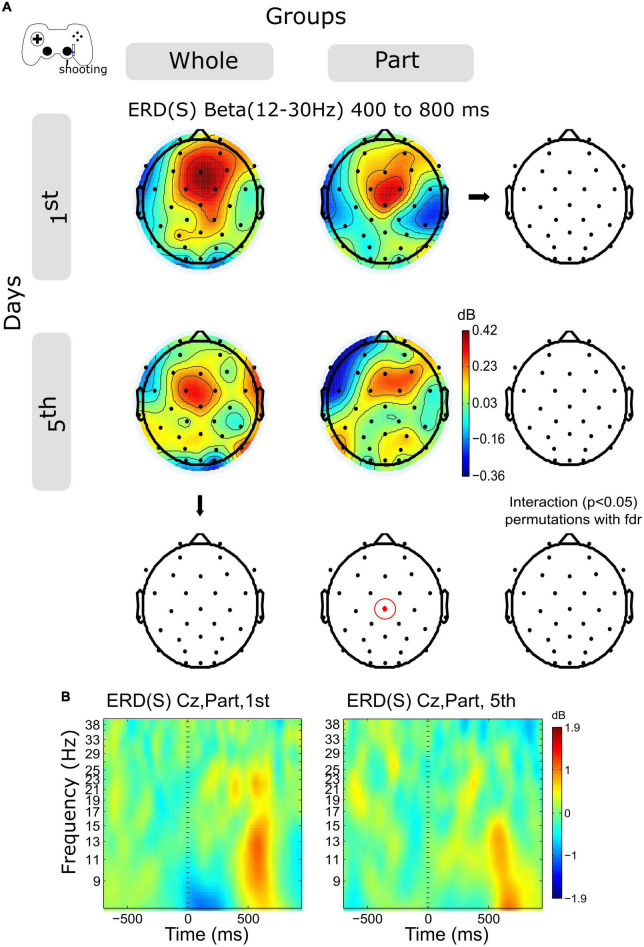
ERD/ERS by days and by groups during shooting. **(A)** The topography of power (dB) in all channels is represented in the scalp map at the time window of 200 to 400 ms. In red dots and red ellipses are depicted the significant differences. **(B)** Cz time-frequency plots of shooting epochs of Part group in days 1st and 5th. Time 0 ms is the motion onset. 1st = baseline test, 5th = integrated test.

The activity of the Beta band ERS (12–30 Hz) shows a decrease in central channels within sessions in both groups (Figure 5A, and Supplementary Figure 2B), however, this variation was significant only for the Part group in channels Cz in the time window 400–800 ms.

## 4. Discussion

In this research, we report the effect of the type of practice (Whole vs. Part) during the adaptation of kinematic and kinetic components of the visuomotor skill required to navigate a video game task. Our published results (Burgos et al., 2018), summarized here, show that after 5 days of training, the simultaneous practice of the kinetic and kinematic components confers an initially higher level of performance than the practice of the same components separately, despite that the amount of practice was equivalent. This difference is explained because the Whole practice allows the optimal integration of the components of the skill, which confers a higher level of performance in the task, especially when precise temporal and spatial coordination between effectors is required. In parallel, the Part practice confronts the challenge of controlling two effectors close in time, a situation that represents a higher cognitive demand. This report analyzes the neural correlates of both types of practice. The analysis of the evoked neural activity by the kinematic component of the skill (advance) reports a significant decrease in neural potential evoked by motor events on the last day of training but at different times depending on the type of practice. A detailed analysis of the temporal evolution of the ERP for the Part group shows that the negative peak is later (about 50 ms) compared to the Whole group and its record on day 1. Such a situation occurs in posterior (related to visual regions) locations. This result suggests that the recruitment of visuomotor neural resources is delayed for the Part group, which must face the challenge of simultaneous effector control (to control both joypad levers), such neural activation occurs with a time lag. This difference in activity peaks may be related to the transition from reactive to anticipatory control and the role of visual and motor regions in implementing such a transition. It is well-documented that during kinematic adaptation tasks, a better adaptation is represented by an anticipatory visuomotor control of the effectors (Sailer, 2005; Mariman et al., 2019). Therefore, the better performance exhibited here by the Whole group, as well as its previously reported visuomotor component, could also be explained by a more advanced anticipatory control (Burgos et al., 2018), which is related to differences in the amplitude and timing of the motor ERP. For the Part group, on day five they must confront the challenge of integrating kinematic and kinetic control. Therefore, the higher cognitive load can also contribute to the reported delayed evoked activity. Looking at the ERP in the later period (50 to 150 ms), we again observe a lower amplitude during the fifth day of training for posterior scalp locations. Such evidence confirms our interpretation regarding the visual contribution to the achievement of advanced levels in a kinematic adaptation. In this case, given that the Whole group exhibits less evoked activity, it may be more specific and/or of lesser magnitude, which would account for a refinement in the selection of neural networks in the course of practice (Della-Maggiore and McIntosh, 2005).

Now, when we observe the activity evoked by the kinetic component (shooting), we detect a positive peak of activity around 200 ms after the movement end (close to 600 ms after the movement onset). This peak was modulated by training so that both types of practice exhibited a lower peak on day 5, being significantly lower for the Whole group. The timing of this positive evoked response resembles the positive component (Pe) of error-related ERP, which has been related to the activity monitoring system associated with the cingulate cortex (Falkenstein et al., 2000). Previous studies have described how the magnitude of the action error modulates its subsequent potential, with a greater response to larger errors, both in pointing tasks using prism goggles (Vocat et al., 2011) and in kinematic adaptations to distortions (Anguera et al., 2009). In our case, we can account for the positive component of this error-related ERP, which, consistent with the studies mentioned above, is lower in the late stage of training, where errors decrease and performance improves. Significantly for the Whole group, Pe modulation occurs at frontal and parietal electrodes. Complementarily, this ERP component has also been associated with the emotional aspects of making mistakes and their conscious assessment (Falkenstein et al., 2000; Vocat et al., 2011).

Furthermore, given its posterior location, it would be associated with spatial visual error detection (Luaute et al., 2009). However, the origin of this Pe is not entirely clear. Beyond these interpretations, this result confirms that the Whole practice confers an advanced state of control, with less recruitment of the monitoring system.

Analyzing the modulation of the spectral activity related to the kinematic component, we observed on the initial day of training a discrete moment of ERS, especially evident for the Part group (Figure 5). Such activity is related to the duration of the advancing movement, which on the initial day was predominantly short (duration close to 200 ms, data not included). Thus, such ERS corresponds to the rebound of activity in the Alpha and Beta range after the movement. Since the duration of the advance was longer on day 5, we see a prolonged ERD, especially in the Beta range. Statistical analysis shows a significant Beta-power decrease in the Part group for a central electrode (FC1), which confirms our previous observation. Thus, at advancing epochs, we observe an evident change in spectral activity indicative of a change in kinematic control. However, such differences can also be explained by the performance of the Part group on day 1, which warns us to view this result with caution. In the case of the Whole group, these results suggest a consolidated strategy from day 1, consisting of the chaining of sub-movements or the prolongation of a single movement. However, such a conclusion must be ratified by a more exhaustive kinematic analysis.

Finally, in the case of shooting, we observe a sequential modulation of the spectral power that transits from the ERD to the ERS. Unlike the advance, in this case, the rebound of activity in the Beta band is smaller for the Part group. Previous studies have related the ERS after the movement with the activation of sensorimotor and frontal circuits associated with the evaluation of the action, the previous history of errors (in the Bayesian framework), and the magnitude of those (Tan et al., 2014), the salience of the error (Torrecillos et al., 2015), as well as learning processes after a long period of practice (Tatti et al., 2021), as in our case. Particularly in our practice design, the Part group faced an unexpected situation based on their training history: the requirement to control two effectors simultaneously. This fact probably modulated the neural processing associated with shooting errors, which, unlike the Whole group, which trained in an integrated manner, the Part group probably represented a salient and unexpected situation. In this way, the decrease of the ERS could index the evaluation and learning of the error. Further analyzes are required to study the association between the magnitude of the error and the spectral activity in the Beta band to corroborate this interpretation.

We acknowledge the limitations of our study. The most important is the small sample size of both groups. This situation probably explains why there are no changes over time for all the parameters of neural activity examined and the non-existence of interaction between the effects. In this way, although we can differentiate the effect of the type of practice, our interpretations would be more robust if we could capture visually detected changes, especially in the related spectral activity between training days. Another limitation is the characterization of the kinematic control in advancing trials. In this case, since there was no restriction to advance, this ability could be achieved through the combination of discrete movements, single movements prolonged in time, or a mixture of both, depending on the challenge imposed by the video game. Then the heterogeneity in the duration of the movement made it difficult to analyze the evoked and spectral activity related to its onset and end of the movement. Additionally, to balance the amount of practice between the Whole and Part groups, we follow the recommendation that the total time of practice should be the same for each group. However, given that the Whole group had the chance to practice shooting and advancing at the same time, the total number of repetitions for advancing or shooting couldn’t be controlled. Finally, given that our study approach relates brain activity to the onset of movement, it was not possible to include moments of bimanual movement due to the difficulty in identifying the origin of the activity. Such movements are the most direct instance of integration of task motor components. However, recognizing this limitation, our data analysis examines such an effect by contrasting it with the integration challenges faced by the Part group on day 5 of training.

In conclusion, the whole practice confers a transient performance advantage related to the simultaneous practice of the kinetic and kinematic components of the task. Concerning changes in neural activity, evoked potential activity (ERP) demonstrates a lower demand for neural resources to solve the task in the late stages of learning. However, the need for integration persists for the part group, which is reflected in the timing of the response. Finally, Beta’s activity related to advancing (ERD) and shooting (ERS) ratifies that the part group faces a control challenge concerning the learning process developed in the late stages of learning.

## Data availability statement

The raw data supporting the conclusions of this article will be available upon request.

## Ethics statement

The study was reviewed and approved by the Comité de Ética en Investigación en Seres Humanos, Facultad de Medicina, Universidad de Chile. The participants provided their written informed consent to take part of this study.

## Author contributions

JM and PB contributed to the conception, design and interpretation of the data, drafting, and revising. TB-M contributed to the analysis, interpretation of data, and critical revision. RG-R contributed to the critical revision. PM contributed to the conception, design, interpretation of the data of the work, and revised. All authors were accountable for all aspects of the work, contributed to the article, and approved the submitted version.

## Footnotes

1
https://www.biosemi.com/faq/shielding%20vs%20active%20electrodes.htm

2
http://sccn.ucsd.edu/eeglab/

3
http://www.nitrc.org/projects/cleanline/

4
http://sccn.ucsd.edu/wiki/Amica

## Supplementary material

Supplementary Figure 1 *P*-values without fdr correction for event-related potentials (ERPs) of advancing and shooting. **(A)** The *p*-values correspond to Figure 2A left (advancing in the time windows 0 to 50 ms). **(B)** Correspond to Figure 2A right (advancing in the time windows 50 to 150 ms). **(C)** Correspond to Figure 3A (shooting in the time windows 580 to 660 ms).

Supplementary Figure 2 *P*-values without fdr correction for ERD/ERS of advancing and shooting. **(A)** The *p*-values correspond to Figure 4A (advancing in the time windows 200 to 400 ms). **(B)** Correspond to Figure 5A (shooting in the time windows 400 to 800 ms).
