# Supplementary material for: Event-related (de)synchronization and potential in whole vs. part sensorimotor learning
Source: Front Syst Neurosci. 2023 Mar 21;17:1045940. doi: 10.3389/fnsys.2023.1045940 (PMC10070693; doi:10.3389/fnsys.2023.1045940)
Supplement: Supplementary file 1 [file Table_1.DOCX]

| Interaction Group-Session/Block | Input Parameters | | Output Parameters | | |
| --- | --- | --- | --- | --- | --- |
|  | Effect size  partial eta squared | Correlation among rep measures | n required | Power with  n=required | **Power with n=18** |
| Overall Advance Figure 1B | 0.140 | 0.629 | 6 | 0.822 | 0.999 |
| Advance  Figure 1D Blocks 131-135 vs 1-5 | 0.122 | 0.309 | 10 | 0.821 | 0.998 |
| Overall Shooting Figure 1C | 0.226 | 0.556 | 6 | 0.954 | 1 |
| Shooting Figure 1E Blocks 131-135 vs 1-5 | 0.050 | 0.540 | 18 | 0.834 | 0.834 |

*Supplementary Table 1, Computations of the power of the results based on effect size using G Power 3.1.9.2 , for F test Family, repeated measures, within-between interaction using alpha = 0.05 and beta = 0.2 for error probability.*
