# Supplementary figures and images for: Event-related (de)synchronization and potential in whole vs. part sensorimotor learning
Source: Front Syst Neurosci. 2023 Mar 21;17:1045940. doi: 10.3389/fnsys.2023.1045940 (PMC10070693; doi:10.3389/fnsys.2023.1045940)

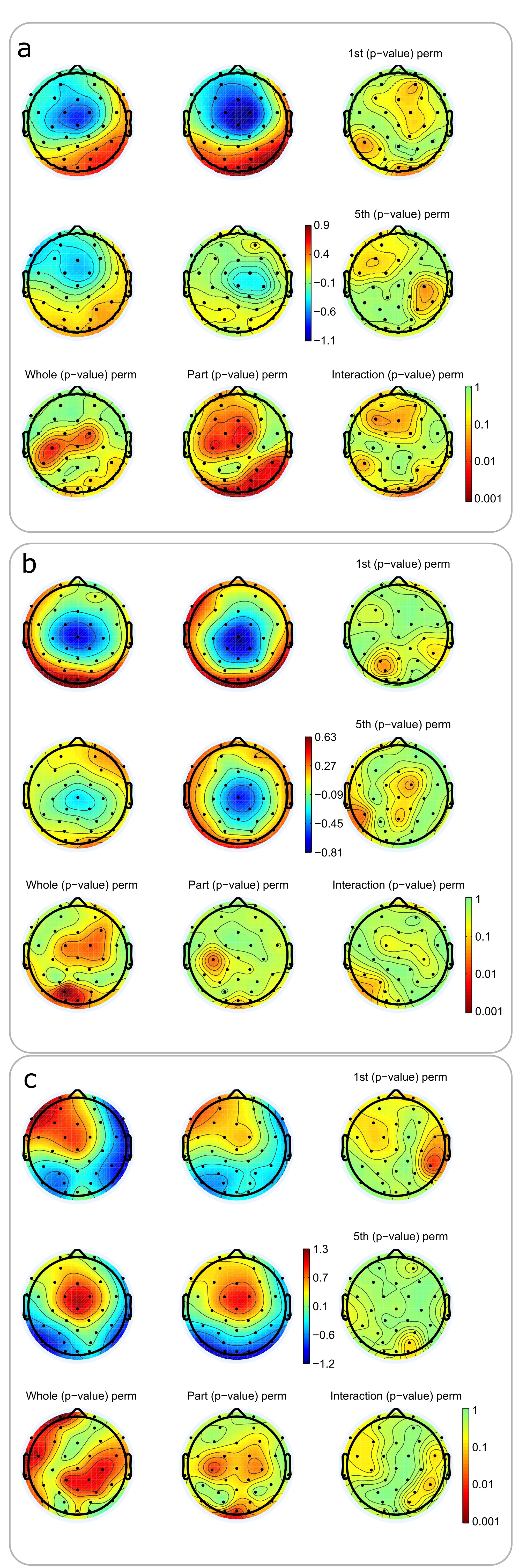

Supplement: Supplementary Figure 1 — P-values without fdr correction for event-related potentials (ERPs) of advancing and shooting. (A) The p-values correspond to Figure 2A left (advancing in the time windows 0 to 50 ms). (B) Correspond to Figure 2A right (advancing in the time windows 50 to 150 ms). (C) Correspond to Figure 3A (shooting in the time windows 580 to 660 ms). [file Image_1.JPEG]

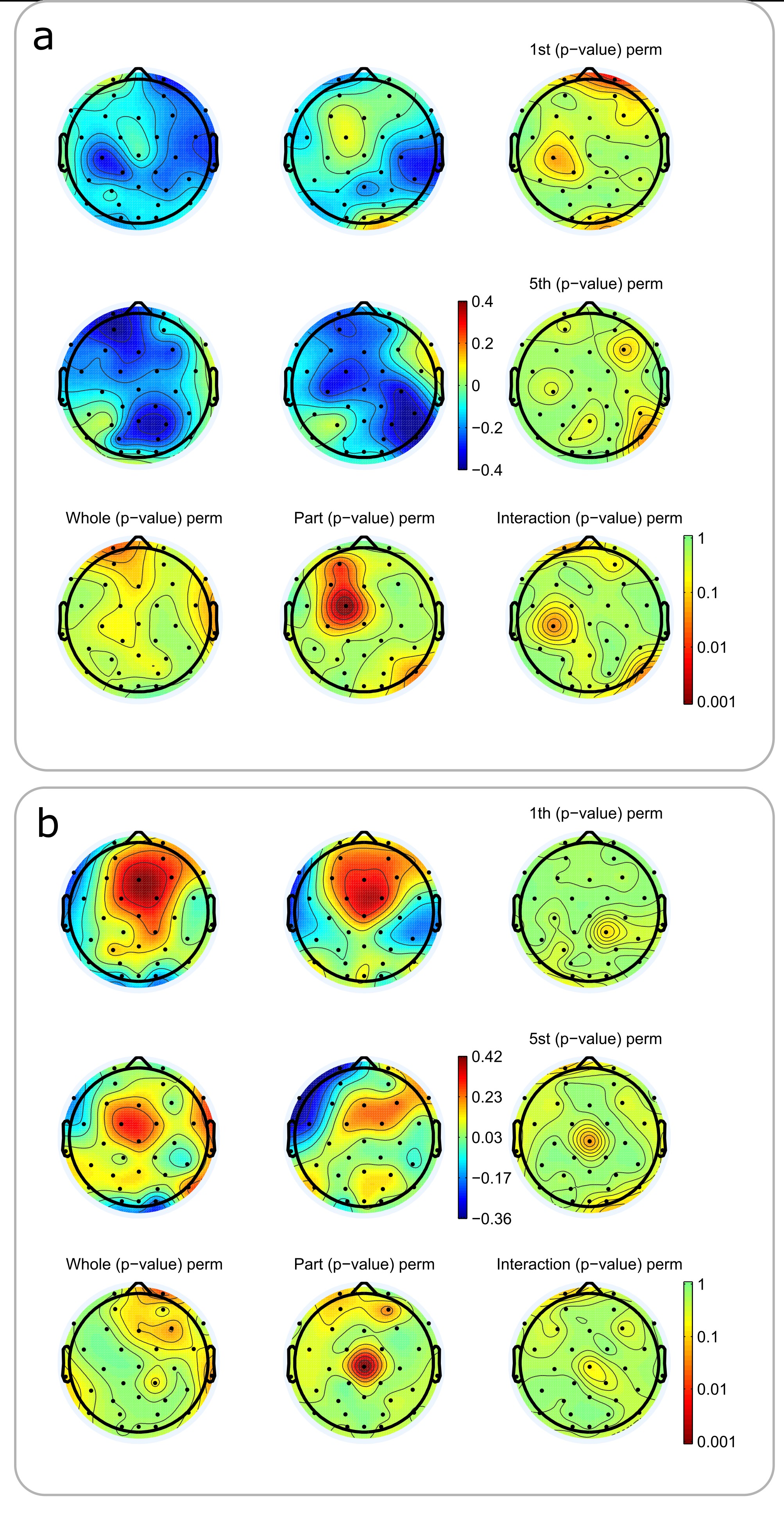

Supplement: Supplementary Figure 2 — P-values without fdr correction for ERD/ERS of advancing and shooting. (A) The p-values correspond to Figure 4A (advancing in the time windows 200 to 400 ms). (B) Correspond to Figure 5A (shooting in the time windows 400 to 800 ms). [file Image_2.JPEG]
